# Longitudinal Changes in Brain Gyrification in Schizophrenia Spectrum Disorders

**DOI:** 10.3389/fnagi.2021.752575

**Published:** 2021-12-24

**Authors:** Tien Viet Pham, Daiki Sasabayashi, Tsutomu Takahashi, Yoichiro Takayanagi, Manabu Kubota, Atsushi Furuichi, Mikio Kido, Kyo Noguchi, Michio Suzuki

**Affiliations:** ^1^Department of Neuropsychiatry, University of Toyama Graduate School of Medicine and Pharmaceutical Sciences, Toyama, Japan; ^2^Research Center for Idling Brain Science, University of Toyama, Toyama, Japan; ^3^Arisawabashi Hospital, Toyama, Japan; ^4^Department of Psychiatry, Graduate School of Medicine, Kyoto University, Kyoto, Japan; ^5^Toyama City Hospital, Toyama, Japan; ^6^Department of Radiology, University of Toyama School of Medicine, Toyama, Japan

**Keywords:** gyrification, local gyrification index, magnetic resonance imaging, schizophrenia, schizotypal disorder, longitudinal, gyrification trajectory

## Abstract

Previous magnetic resonance imaging (MRI) studies reported increased brain gyrification in schizophrenia and schizotypal disorder, a prototypic disorder within the schizophrenia spectrum. This may reflect deviations in early neurodevelopment; however, it currently remains unclear whether the gyrification pattern longitudinally changes over the course of the schizophrenia spectrum. The present MRI study using FreeSurfer compared longitudinal changes (mean inter-scan interval of 2.7 years) in the local gyrification index (LGI) in the entire cortex among 23 patients with first-episode schizophrenia, 14 with schizotypal disorder, and 39 healthy controls. Significant differences were observed in longitudinal LGI changes between these groups; the schizophrenia group exhibited a progressive decline in LGI, predominantly in the fronto-temporal regions, whereas LGI increased over time in several brain regions in the schizotypal and control groups. In the schizophrenia group, a greater reduction in LGI over time in the right precentral and post central regions correlated with smaller improvements in negative symptoms during the follow-up period. The cumulative medication dosage during follow-up negatively correlated with a longitudinal LGI increase in the right superior parietal area in the schizotypal group, but did not affect longitudinal LGI changes in the schizophrenia group. Collectively, these results suggest that gyrification patterns in the schizophrenia spectrum reflect both early neurodevelopmental abnormalities as a vulnerability factor and active brain pathology in the early stages of schizophrenia.

## Introduction

Brain gyrification is regarded as an early neurodevelopmental marker of cortical complexity because its pattern is genetically mediated (Bartley et al., 1997) and it undergoes the greatest development during pregnancy but remains rather stable after birth (Armstrong et al., 1995; Zilles et al., 2013). Previous magnetic resonance imaging (MRI) studies demonstrated a widespread brain hypergyrification pattern in the early stages of schizophrenia (Schultz et al., 2013; Nanda et al., 2014; Sasabayashi et al., 2017a), which potentially reflects an early neurodevelopmental pathology, including white matter maldevelopment and aberrant neural connectivity (Sasabayashi et al., 2021). Increased brain gyrification in the frontal and other cortical regions was also detected in individuals with genetic (Falkai et al., 2007; Neilson et al., 2018) and clinical risk for the development of psychosis (Tepest et al., 2013; Sasabayashi et al., 2017b) and in patients with schizotypal disorder (Sasabayashi et al., 2020), who share biological commonalities with schizophrenia patients as part of the schizophrenia spectrum in the context of the neurodevelopmental hypothesis but are mostly spared from developing overt psychosis (Siever and Davis, 2004). These findings support brain hypergyrification being an early developmental trait marker associated with vulnerability to psychosis (Sasabayashi et al., 2021).

Gyrification was previously reported to be normal (Haukvik et al., 2012) or even decreased (Nesvåg et al., 2014; Palaniyappan and Liddle, 2014) in patients with schizophrenia, particularly in chronic cases (reviewed by Harris et al., 2007; Nanda et al., 2014; Sasabayashi et al., 2021). In addition to the potential influence of different sample characteristics (e.g., clinical subtypes, Sallet et al., 2003; Takayanagi et al., 2019, and treatment responses; Palaniyappan et al., 2013b) on the gyrification pattern in schizophrenia, these discrepancies may partly be attributed to factors associated with illness stages, such as illness chronicity and antipsychotic medication (Tomelleri et al., 2009). A 2-year follow-up MRI study on first-episode schizophrenia (Palaniyappan et al., 2013a) reported a longitudinal decline in gyrification, predominantly in the fronto-temporo-limbic regions, which may be associated with treatment responses (Nelson et al., 2020). Since the cortical surface may flatten during adolescence as a late neurodevelopmental process (Alemán-Gómez et al., 2013), this longitudinal decline appears to be consistent with the notion that a progressive brain pathology in the early stages of psychosis is associated with the acceleration of normal brain maturational processes (Pantelis et al., 2007). However, these longitudinal studies only examined selected brain regions (Palaniyappan et al., 2013a) or average gyrification per hemisphere (Nelson et al., 2020). Furthermore, to the best of our knowledge, potential changes in gyrification over time have not yet been investigated in schizotypal patients using MRI.

Therefore, the present MRI study investigated potential longitudinal changes in the brain gyrification pattern in the entire cortex among patients with first-episode schizophrenia, those with schizotypal disorder, and matched healthy controls. Based on the gyrification patterns observed at different illness stages of schizophrenia and previous longitudinal findings (Palaniyappan et al., 2013a), we hypothesized that patients with first-episode schizophrenia exhibit a greater longitudinal decline in gyrification than healthy controls. We also predicted that the brain gyrification pattern is more stable in patients with schizotypal disorder who are vulnerable to psychosis, but do not exhibit active brain changes (Takahashi and Suzuki, 2018). Furthermore, we investigated whether potential longitudinal changes in brain gyrification in the patient groups are associated with medication and changes in clinical symptoms.

## Materials and Methods

### Subjects

Subjects comprised 23 patients with first-episode schizophrenia, 14 with schizotypal disorder, and 39 healthy controls (Table 1), all of whom were right-handed and physically healthy and had no previous history of head trauma, neurological illness, substance abuse disorder, or serious medical disease. In the longitudinal brain assessment, all subjects were scanned twice during clinical follow-up (inter-scan interval; mean = 2.7 years, *SD* = 0.6 years) using the same MR scanner and parameters. Although this was the first follow-up study of local gyrification index (LGI) changes in our sample, we previously assessed cross-sectional LGI differences in larger cohorts (Sasabayashi et al., 2017a,^2020^) that included some of the subjects in the present study (18/23 patients with first-episode schizophrenia, 8/14 with schizotypal disorder, and 17/39 healthy controls). The Committee on Medical Ethics of Toyama University approved the present study. All subjects provided their written informed consent following a detailed explanation of the study.

**TABLE 1:** Subjects characteristics in the present study.

	HC (*N* = 39)	SzTypal (*N* = 14)	Sz (*N* = 23)	Group comparisons
Male/female	22/17	10/4	15/8	Chi-square = 1.144, *p* = 0.564
Age at baseline scan (years)	24.6 (4.7)	23.0 (4.9)	23.5 (4.8)	*F*(2, 73) = 0.788, *p* = 0.458
Height (cm)	165.8 (8.0)	166.6 (9.2)	165.0 (7.9)	*F*(2, 73) = 0.169, *p* = 0.845
Education (years)	15.6 (2.1)	12.5 (2.4)	13.1 (1.6)	*F*(2, 73) = 17.218, *p* < 0.001; Sz, SzTypal < C
Parental education (years)^a^	13.1 (2.4)	12.1 (1.6)	12.7 (2.1)	*F*(2, 73) = 0.947, *p* = 0.393
Inter-scan interval (years)	2.5 (0.4)	2.9 (0.8)	2.6 (0.8)	*F*(2, 73) = 1.867, *p* = 0.162
Onset age (years)	–	–	22.4 (4.9)	–
Illness duration at baseline (months)	–	–	9.5 (9.1)	–
Medication dose (haloperidol equivalent mg)				
Dose at baseline (mg/day)	–	6.4 (7.6)	13.5 (11.4)	*F*(1, 35) = 4.331, *p* = 0.045; SzTypal < Sz
Cumulative dose during follow-up (mg)	–	7030.7 (7243.2)	9526.4 (8609.2)	*F*(1, 35) = 0.820, *p* = 0.371
Duration of medication at baseline (months)	–	42.3 (60.2)	7.8 (9.7)	*F*(1, 35) = 7.379, *p* = 0.010; Sz < SzTypal
Total SAPS score^a^				
Baseline	–	17.6 (9.6)	29.0 (24.3)	*F*(1, 31) = 2.572, *p* = 0.119
Follow-up	–	13.6 (11.3)	17.0 (17.1)	*F*(1, 34) = 0.452, *p* = 0.506
Total SANS score^a^				
Baseline	–	54.8 (22.1)	52.1 (25.5)	*F*(1, 31) = 0.099, *p* = 0.755
Follow-up	–	42.3 (16.6)	38.0 (22.5)	*F*(1, 34) = 0.376, *p* = 0.544
**Data are shown as means (SD).**				

*^a^Data were missing for 4 patients (1 schizotypal and 3 schizophrenia patients) at baseline and for 1 schizophrenia patient in the follow-up. HC, healthy controls; SANS, scale for the assessment of negative symptoms; SAPS, scale for the assessment of positive symptoms; Sz, schizophrenia; SzTypal, schizotypal disorder.*

As previously described (Suzuki et al., 2005; Takahashi et al., 2010), patients with first-episode schizophrenia and schizotypal disorder who fulfilled the ICD-10 research criteria (World Health Organization., 1993) were recruited from the inpatient and outpatient clinics of the Department of Neuropsychiatry of Toyama University Hospital. Briefly, first-episode patients were defined as those with an illness duration of ≤ 1 year or those experiencing psychiatric hospitalization for the first time during baseline scanning. All schizotypal patients also met the DSM Axis II diagnosis of schizotypal personality disorder (American Psychiatric Association., 1994) and did not develop overt psychosis during the follow-up period. The Scale for the Assessment of Negative Symptoms (SANS) (Andreasen, 1983) and the Scale for the Assessment of Positive Symptoms (SAPS) (Andreasen, 1984) were used to rate clinical symptoms at the time of baseline and follow-up scanning. During the follow-up period between scans, 13 patients (7 schizophrenia and 6 schizotypal patients; typical group) were predominantly treated with typical antipsychotics or received substantial amounts of both typical and atypical ones, while 24 patients (16 schizophrenia and 8 schizotypal patients; atypical group) were treated mostly with atypical antipsychotics.

Members of the community, hospital staff, and university students were recruited as healthy controls. A questionnaire with 15 items related to personal (13 items, e.g., a previous history of traumatic head injury, seizures, neurological or psychiatric diseases, hypothyroidism, hypertension, diabetes mellitus, complications of pregnancy, and substance use) and family (2 items) histories of illness was completed, which revealed no personal or family history of psychiatric illness among first-degree relatives.

### Image Acquisition

Subjects were scanned using 1.5-T Magnetom Vision (Siemens Medical System, Inc., Erlangen, Germany) with the three-dimensional gradient-echo sequence FLASH (fast low-angle shots) yielding 160–180 contiguous T1-weighted slices with a thickness of 1.0 mm in the sagittal plane. Imaging parameters were as follows: repetition time = 24 ms; echo time = 5 ms; flip angle = 40; field of view = 256 mm; and matrix size = 256 × 256 pixels. The voxel size was 1.0 × 1.0 × 1.0 mm^3^.

### Imaging Processing

FreeSurfer software (version 5.3^1^) preprocessed T1-weighted images based on a standard auto-reconstruction algorithm using the normalization of non-uniform intensities, non-brain tissue removal, affine registration to the Montreal Neurological Institute (MNI) space and Talairach transformation, and the segmentation of gray/white matter tissue (Fischl, 2012). A visual inspection of reconstructed images followed by the manual editing of any inaccuracies in tissue segmentation was performed by one trained investigator (TVP) blinded to subjects’ identities.

Based on the pial surface reconstruction, an algorithm for measuring 3D LGI at each vertex across each hemisphere was performed (Sasabayashi et al., 2017a,b). Details of the LGI computation may be found in the validation paper (Schaer et al., 2008) and at https://surfer.nmr.mgh.harvard.edu/fswiki/LGI. LGI changes over time were assessed using the standard and automated FreeSurfer longitudinal pipelines based on a within-subject template estimation (Reuter et al., 2012). Specifically, an unbiased within-subject template (Reuter and Fischl, 2011) was created for robust and inverse consistent registration (Reuter et al., 2010). Several processing steps, such as skull stripping, Talairach transformation, atlas registration, spherical surface mapping, and parcellation, were then initialized using common information from the within-subject template, which significantly increased reliability and statistical power (Reuter et al., 2012).

### Statistical Analysis

Demographic and clinical differences between groups were examined by the chi-squared test or a one-way analysis of variance (ANOVA).

All vertex-wise LGI values were individually mapped on a common spherical coordinate system (fsaverage) that was smoothed with a 5-mm Gaussian kernel. Regarding cross-sectional baseline group comparisons as well as longitudinal comparisons in each group (baseline vs. follow-up), LGI values at each vertex were investigated using a general linear model with age, sex, the duration of medication, and medication dosage as covariates, with the Query Design Estimate Contrast application in FreeSurfer software creating contrasts. Concerning pairwise group comparisons of longitudinal LGI changes, a longitudinal two-stage model was employed to examine LGI changes across groups with age, sex, the inter-scan interval, and cumulative medication dosage during follow-up period as covariates. In the first stage, we performed a linear fit for each subject independently to reduce the repeated measures to a single number. In the second stage, a regular cross-sectional analysis was conducted across subjects. For the medication type, we compared longitudinal LGI changes between the patients with typical and atypical subgroups using the same model. We conducted a vertex-by-vertex correlation analysis between baseline LGI and cumulative medication dosage at baseline (mean = 529.2 mg, *SD* = 535.2 mg) in the schizophrenia group using a general linear model controlling for age and sex. The same model controlling for age, sex, and the inter-scan interval was used in the vertex-by-vertex correlation analyses between longitudinal LGI changes in patients and the cumulative medication dosage during follow-up, or symptom changes (SAPS/SANS scores in the follow-up—SAPS/SANS scores at baseline). The cumulative medication dosage during follow-up was also added as a covariate for interactions with symptom changes. To correct for multiple comparisons, a Monte Carlo simulation implemented in the AlphaSim program of Analysis of Functional NeuroImages (AFNI) was used in these analyses (Hagler et al., 2007). To define significant clusters, 10,000 iterations of the Monte Carlo simulation were conducted in each comparison using a threshold of *p* < 0.05.

## Results

### Demographic Backgrounds

Although groups were matched for age, sex, height, and parental education, the control group had a higher level of education than the patient groups (Table 1). The schizophrenia group had a higher SAPS score than the schizotypal group, particularly at baseline. The duration of medication at baseline was significantly longer and the medication dosage at baseline was significantly higher in the schizotypal group than in the schizophrenia group (Table 1).

### Baseline Group Comparisons of Local Gyrification Index

**FIGURE 1:**
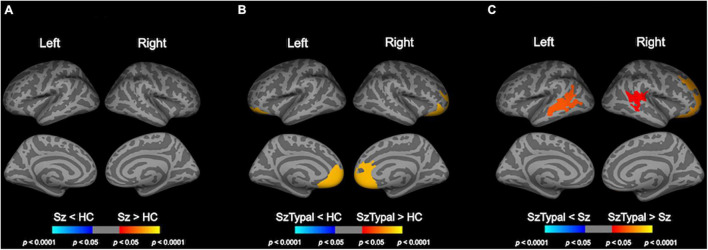
Cortical statistical maps of baseline local gyrification index (LGI) comparisons. The schizophrenia (Sz) group showed no significant LGI changes from the healthy control (HC) group **(A)**. LGI in the bilateral superior frontal, medial orbitofrontal, frontal pole, and rostral anterior cingulate regions was higher in the schizotypal (SzTypal) group than in the HC group **(B)**. LGI in the bilateral superior and medial temporal, right superior, and lateral orbitofrontal regions was higher in the SzTypal group than in the Sz group **(C)**.

Baseline group comparison revealed LGI in the bilateral frontal regions was higher in the schizotypal group, but not in the schizophrenia group, than in the control group (Figure 1). LGI in the bilateral fronto-temporal regions was also higher in the schizotypal group than in the schizophrenia group (Figure 1). There were no significant differences in baseline LGI between the combined patient group (schizophrenia and schizotypal group) and control group.

### Longitudinal Local Gyrification Index Changes in Each Group (Baseline vs. Follow-up)

No cluster exhibited significant LGI changes between baseline and follow-up scans in any diagnostic group after cluster-wise correction.

We also conducted supplementary uncorrected analyses using age, sex, and medication as nuisance variables in order to examine general tendency of LGI changes over time (i.e., increase or decrease); LGI decreased predominantly in the fronto-temporal areas in the schizophrenia group, while it increased in several brain regions in the schizotypal and control groups (Supplementary Figure 1). A similar progressive decline in the fronto-temporal LGI was observed in the combined patient group (Supplementary Figure 2).

### Pairwise Group Comparisons of Longitudinal Local Gyrification Index Changes

**FIGURE 2:**
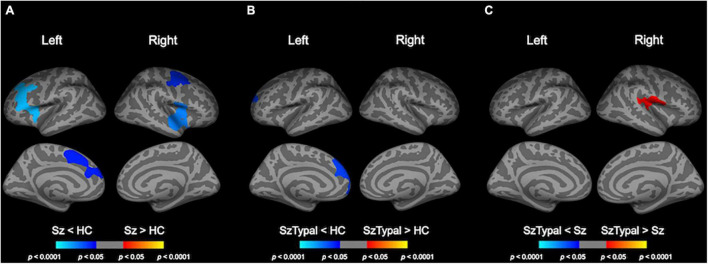
Pairwise group comparisons of longitudinal local gyrification index (LGI) changes. Cortical statistical maps showed that the schizophrenia (Sz) group exhibited a significantly greater decline in LGI over time than the healthy control (HC) group in the caudal middle frontal gyrus, superior frontal gyrus, and pars opercularis gyrus bilaterally, in addition to the rostral middle frontal gyrus, pars triangularis gyrus, and caudal anterior cingulate cortex in the left hemisphere, and superior temporal gyrus, precentral gyrus in the right hemisphere **(A)**. The progressive increase in LGI in the left superior frontal area was smaller in the schizotypal (SzTypal) group than in the HC group **(B)**. The decline in LGI in the right pre- and post-central gyrus, supramarginal gyrus, and transverse temporal gyrus was greater in the Sz group than in the SzTypal group **(C)**.

The progressive decline in LGI, predominantly in the fronto-temporal and parietal regions, was significantly greater in the schizophrenia group than in the control group (Table 2 and Figure 2). There were no regions in which schizophrenia patients had a significant LGI increase compared with healthy control subjects. Although a progressive increase in LGI was observed in the schizotypal and control groups (Supplementary Figure 1), this change in gyrification was significantly greater in the left superior frontal region in the control group (Figure 2). The mean LGI values of these clusters with significant group differences are summarized in Supplementary Table 1. In addition, the combined patient group exhibited progressive LGI decline similar to that observed in schizophrenia group (Supplementary Table 2 and Supplementary Figure 3).

**TABLE 2:** Clusters showing significant differences in pairwise group comparisons of longitudinal local gyrification index changes.

Cluster no.	Cluster size (mm_2_)	Cluster-wise *p*	MNI coordinates			Annotation
			x	y	z	
**Sz < HC**						
1	3634.8	0.0001	–21.3	31.3	28.4	Left caudal and rostral middle frontal, pars triangularis, pars opercularis gyrus, insular cortex
2	1998.1	0.0164	–10.8	19.5	35.8	Left superior frontal gyrus, caudal and rostral anterior cingulate cortex
3	3065.4	0.0006	55.3	–7.0	–7.6	Right superior temporal, precentral, pars opercularis gyrus
4	2304	0.0097	36.4	–4.5	45.7	Right precentral, superior frontal, caudal middle frontal gyrus
**SzTypal < HC**						
5	2348.2	0.0055	–8.6	48.7	23.9	Left superior frontal gyrus, rostral middle frontal, medial orbitofrontal cortex, frontal pole
**Sz < SzTypal**						
6	1707.8	0.0435	43.5	–18.8	17.9	Right pre- and postcentral, supramarginal, transverse temporal gyrus

*HC, healthy controls; Sz, schizophrenia; SzTypal, schizotypal disorder.*

When we included the education, parental education, or illness duration at baseline as one of the controlling factors (illness duration = 0 for the schizotypal and healthy groups), significant clusters similar to the original analyses were observed even after controlling for the parental education and illness duration (Supplementary Figures 4, 5). There was no group difference in the longitudinal LGI changes when we used the education as a covariate (Supplementary Figure 6).

Whereas the schizophrenia subgroup treated with typical antipsychotics (*n* = 7) exhibited progressive LGI decline in the bilateral frontal regions, LGI was declined in the bilateral temporal and left parietal regions for those with atypical antipsychotics (*n* = 16) (Supplementary Figure 7). There was no significant difference in the longitudinal LGI changes between the schizotypal subgroup with typical (*n* = 6) and atypical (*n* = 8) antipsychotics.

### Correlation Analysis of Baseline Local Gyrification Index and Cumulative Medication Dosage at Baseline

In the schizophrenia group, baseline LGI was not related to the cumulative medication dosage at baseline.

### Correlation Analysis of Longitudinal Local Gyrification Index Changes and Clinical Variables

**FIGURE 3:**
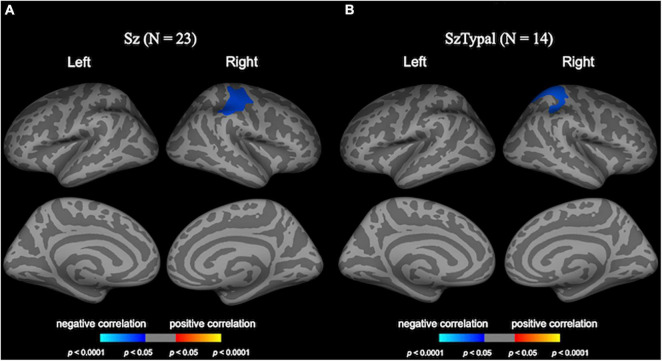
Correlation analyses of longitudinal local gyrification index (LGI) changes and clinical variables. Cortical statistical maps revealed the longitudinal LGI reductions in the right pre/post-central regions negatively correlated with changes in negative symptoms in the schizophrenia (Sz) group **(A)**. The longitudinal LGI increase in the right superior parietal region in the schizotypal (SzTypal) group negatively correlated with the cumulative medication dosage during follow-up **(B)**.

In the schizophrenia group, the greater progressive decline in LGI in the right pre- and post-central regions was associated with smaller improvements in negative symptoms (Figure 3).

The cumulative medication dosage during follow-up negatively correlated with longitudinal LGI changes in the right superior parietal region in the schizotypal group (Figure 3), whereas medication was not associated with longitudinal LGI changes in the schizophrenia group.

## Discussion

To the best of our knowledge, this is the first MRI study to examine and compare longitudinal changes in brain gyrification in the entire cortex among patients with first-episode schizophrenia, those with schizotypal disorder, and healthy controls. The results obtained demonstrated a greater progressive decline in LGI in the fronto-temporal and parietal regions in the schizophrenia group, but not in the schizotypal group, than in the control group, which was partly associated with smaller improvements in negative symptoms after the first episode of the illness. Among schizophrenia spectrum disorders, these changes appear to reflect active brain changes specifically observed in the early stages of schizophrenia, which are also associated with early treatment responses.

Although the formation of the brain sulcogyral pattern is mostly complete by the late second to third trimester (Armstrong et al., 1995; Zilles et al., 2013), healthy subjects may exhibit mild age-related GI changes after birth; the entire cortical GI increases by approximately 20% in the first 2 years after birth, but slowly decreases thereafter (approximately 1%/year during adolescence) (Raznahan et al., 2011; Cao et al., 2017). Although gyral formation, which is considered to be mediated by genetic factors (Bartley et al., 1997), may reflect pre- and perinatal brain development (including early axonal connections, neuronal growth and differentiation, glial proliferation, and synaptogenesis) (Toro and Burnod, 2005), the mechanisms underlying the decline in GI after birth have not yet been elucidated (Cao et al., 2017). However, age-related cortical flattening (i.e., cortical thinning in combination with a decrease in GI) during adolescence may be attributed to synaptic pruning and the effects of the maturation of white matter as a process of late neurodevelopment (Alemán-Gómez et al., 2013). The present finding of no significant longitudinal LGI changes in healthy subjects in early adulthood suggests that this maturation process predominantly occurs at earlier ages and/or gyrification changes after birth are subtle and undetectable in a small sample.

In cross-sectional comparisons, we replicated our previous findings in a larger cohort (Sasabayashi et al., 2020) of schizotypal patients exhibiting an increase in LGI predominantly in the prefrontal regions, which was also reported in the early stages of schizophrenia (Narr et al., 2004; Tepest et al., 2013) as well as in genetic (Falkai et al., 2007; Neilson et al., 2018) and clinical (Tepest et al., 2013; Sasabayashi et al., 2017b) high risk subjects for the development of psychosis. Although hypergyrification was not detected in the schizophrenia group in the present study, which may have been due to its small sample size, we previously demonstrated a widespread brain hypergyrification pattern in an expanded cohort of first-episode schizophrenia patients (*N* = 62) (Sasabayashi et al., 2017a). As described above, the brain gyrification pattern in humans largely depends on early neurodevelopmental processes, such as axonal connections and cortical maturation (Zilles et al., 2013). When taken together with previous findings suggesting that altered connectivity in frontal regions was common under specific clinical conditions (e.g., schizotypal, Nakamura et al., 2005; Hazlett et al., 2012, first-episode schizophrenia; Pettersson-Yeo et al., 2011; Li et al., 2017, and genetic and clinical high risk subjects, Shim et al., 2010; Yoon et al., 2015), our cross-sectional observations of frontal hypergyrification in the schizophrenia spectrum indicate that it is a vulnerability factor associated with aberrant early neurodevelopment. Direct comparison between the schizophrenia and schizotypal groups showed a significantly higher LGI in the prefrontal and temporal regions in the schizotypal group, while our previous study in a larger cohort suggested a higher frontal LGI in the schizophrenia than in the schizotypal patients (Sasabayashi et al., 2020). Thus, cross-sectional difference in LGI between these disorders and potential influencing factors (e.g., medication, clinical symptoms) should be further investigated in an independent cohort.

Consistent with previous longitudinal LGI studies including older participants (Palaniyappan et al., 2013a; Nelson et al., 2020), we observed a greater progressive decline in LGI in the first-episode schizophrenia group than in the control group, which may partly explain the discrepant gyrification findings at different stages of schizophrenia (e.g., normal or even hypogyrification in chronic schizophrenia; reviewed by Harris et al., 2007; Nanda et al., 2014). These progressive changes in LGI were specific to the schizophrenia group and were not observed in the schizotypal group, suggesting that the active brain processes associated with the decline in LGI partly explain the neural mechanisms underlying the onset of psychosis. We further demonstrated that lower educational attainment has significant influence on longitudinal LGI changes in schizophrenia, partly supporting the notion that general cognitive impairments reflect core features of schizophrenia which are evident throughout the course of the illness (Ohi et al., 2018). Likewise, a greater LGI decline of the temporo-parietal areas in the schizophrenia group than in the schizotypal group might be partly explained by progressive gray matter reduction of the corresponding regions specific to the schizophrenia patients, which may underlie positive psychotic symptoms (Takahashi et al., 2010). Based on the potential relationship between age-related changes in gyrification and late neurodevelopmental processes (e.g., synaptic pruning and myelination) in healthy subjects (Alemán-Gómez et al., 2013), the present results provide support for the hypothesis that overmaturation during adolescence and young adulthood plays a role in the development of psychosis (Pantelis et al., 2007). However, the functional significance of our longitudinal LGI results warrants further study in combination with other modalities that assess brain function/connectivity (Palaniyappan and Liddle, 2014).

One major result of the present study was the correlation between the decline in LGI in the pre-/post-central region and smaller improvements specifically in negative symptoms during the first episode of schizophrenia, which appears to support a role for functional/connectivity deficits involving this brain region in negative symptomatology in the early stages of schizophrenia (Li et al., 2015; Vanes et al., 2019). This is also consistent with findings by Palaniyappan et al. (2013a,b) showing that a decline in LGI at baseline and longitudinal reductions in LGI in first-episode schizophrenia predicted poor treatment responses and cognitive deficits; however, this relationship was detected at the fronto-insular regions. Interestingly, our previous cross-sectional study suggested LGI decline with illness chronicity in similar brain regions only for the subgroup of schizophrenia who had no persistent negative symptoms (i.e., non-deficit type) (Takayanagi et al., 2019). However, it remains unknown why longitudinal LGI changes after the onset of schizophrenia can contribute to negative but not positive symptomatology. Furthermore, a recent longitudinal study on unmedicated schizophrenia (Nelson et al., 2020) demonstrated that the decline in LGI in the fronto-parietal regions (including the precentral gyrus) was related to a “better” treatment response. As clinical symptoms of schizophrenia can persist only in some cases and can be reversibly altered, the associations of treatment responses with longitudinal LGI changes should be interpreted with caution. In addition, although the exact reasons for these discrepancies remain unclear, potential medication effects may be a contributing factor. Although a relationship was not observed between the longitudinal decline in LGI and medication in the schizophrenia group, the cumulative medication dosage during follow-up in the schizotypal group negatively correlated with changes in LGI in the parietal region. Since the schizophrenia group was previously treated for a substantial period on average at baseline, antipsychotic medication may have affected their symptom severity and LGI changes. Although the lack of longitudinal data of cognitive functioning did not enable us to test the hypothesis, our findings of a progressive LGI decline predominantly in the fronto-temporal regions, corresponding to the language-related regions around the Sylvian fissure, might be implicated in the production and perception of speech. Indeed, individuals with high-risk for psychosis and first-episode/chronic schizophrenia patients were reported to share an impaired verbal fluency with progression according to illness stages (Henry and Crawford, 2005), which may be associated with a decline in the fronto-insular gyrification (Palaniyappan et al., 2013a). Therefore, further studies are needed to clarify the potential factors affecting changes in gyrification during the course of schizophrenia.

There are a number of limitations that need to be addressed. Since a large number of patients withdrew from the clinical follow-up, the sample size of the present study was small. The unequal sample size with very few schizotypal patients would also affect the statistical power for the comparisons across the three groups. The LGI findings in the combined patient group (schizophrenia and schizotypal disorder) were largely the same as those in schizophrenia, which could be due to small sample size of the schizotypal group. We did not replicate baseline frontal hypergyrification in patients with first-episode schizophrenia (Sasabayashi et al., 2017a) in the present study, which may have been also due to insufficient statistical power. Furthermore, although illness chronicity may affect the gyrification pattern differently depending on the subtypes of schizophrenia (Takayanagi et al., 2019), we were unable to examine this heterogeneity due to the small sample size. Therefore, future longitudinal studies with a larger and well-defined cohort in various illness stages (e.g., before and after the onset of psychosis, chronic stages) are needed to evaluate the changes in gyrification pattern and its linkage with clinical manifestation (e.g., positive or negative symptom change and cognitive decline) during the course of the schizophrenia spectrum. If the LGI findings (especially longitudinal changes at early stages) in schizophrenia spectrum are associated with later illness course (i.e., symptom severity, treatment response, and cognitive deficits), they may have a clinical utility as a predictive marker of outcome. Further, the possibility exists that longitudinal LGI changes may be a target of treatment to prevent illness chronicity, while it is unknown whether early intervention could ameliorate these LGI changes. Another limitation, as described above, is that all patients both in the schizophrenia and schizotypal group had been treated with antipsychotics even at the baseline (median = 3.0 months), which may have biased the results obtained and made it difficult to identify the pathophysiological characteristics of the two groups. Our preliminary results suggested different effects of the type of antipsychotics (typical vs. atypical) on longitudinal LGI changes in schizophrenia, but such effects as well as potential effects of other psychotropic agents (e.g., benzodiazepines, mood stabilizers) should be further tested in a larger cohort. Moreover, since an altered gyrification pattern has been reported in many neuropsychiatric disorders (e.g., mood disorders, Nenadic et al., 2015; Han et al., 2017, and autism spectrum disorder; Kohli et al., 2019), further transdiagnostic comparisons of the trajectories of gyrification are needed to clarify the disease specificity of the present results and their role in the pathophysiology of the schizophrenia spectrum. Lastly, although a multimodal neuroimaging study demonstrated the topographical overlapping between LGI and degree centrality changes (Palaniyappan and Liddle, 2014), it is still elusive whether gyrification patterns implicate connectional characteristics of corresponding regions. Confirming the potential relationship between LGI and functional connectivity measures may be useful for interpreting the present findings.

## Conclusion

In conclusion, combined with our previous cross-sectional findings from larger cohorts (Sasabayashi et al., 2017a,^2020^), the present results suggest that the brain gyrification pattern in the schizophrenia spectrum reflects both early and late neurodevelopmental pathologies. Increases in LGI predominantly in the prefrontal regions, which were commonly observed in the schizophrenia and schizotypal groups, may represent a static vulnerability factor associated with aberrant early neurodevelopment, whereas the progressive decrease in LGI in the fronto-temporal and parietal regions specifically observed during the first episode of schizophrenia appears to reflect an exaggeration of brain maturation during adolescence.

## Data Availability Statement

The datasets utilized for this article are not available immediately because we do not have permission to share them. Requests to access the datasets should be directed to corresponding author.

## Ethics Statement

The studies involving human participants were reviewed and approved by the Committee on Medical Ethics of Toyama University. Written informed consent to participate in this study was provided by all participants and the legal guardian/next of kin of participants under 20 years old.

## Author Contributions

In this study, MS and TT conceived the idea and design of this study. DS, YT, AF, and MKi recruited subjects and were involved in clinical assessments. TVP and DS preprocessed the MRI data and conducted statistical analyses. MKu and KN provided technical support for MRI scanning and data processing. TVP, DS, TT, and MS interpreted the results. TVP wrote the manuscript. DS, TT, and MS contributed to the writing and editing of the manuscript. All authors contributed to and approved the final manuscript.

## Conflict of Interest

The authors declare that the research was conducted in the absence of any commercial or financial relationships that could be construed as a potential conflict of interest.

## Publisher’s Note

All claims expressed in this article are solely those of the authors and do not necessarily represent those of their affiliated organizations, or those of the publisher, the editors and the reviewers. Any product that may be evaluated in this article, or claim that may be made by its manufacturer, is not guaranteed or endorsed by the publisher.

## Footnotes

1
http://surfer.nmr.mgh.harvard.edu/
