# Supplementary material for: Longitudinal Changes in Brain Gyrification in Schizophrenia Spectrum Disorders
Source: Front Aging Neurosci. 2021 Dec 24;13:752575. doi: 10.3389/fnagi.2021.752575 (PMC8739892; doi:10.3389/fnagi.2021.752575)

Supplementary Material

Supplementary Table 1. Absolute local gyrification index (LGI) values in each time point and percent LGI change at the clusters showing significant differences in pairwise group comparisons of longitudinal LGI changes.
Supplementary Table 2. Absolute local gyrification index (LGI) values in each time point and percent change at the clusters showing significant differences in pairwise group comparisons of longitudinal changes between combined patient group and healthy controls.
Supplementary Figure 1 Longitudinal local gyrification index (LGI) changes in each group (baseline *vs.* follow-up) using uncorrected statistical analyses to visualize the direction of changes.
Supplementary Figure 2 Longitudinal local gyrification index (LGI) changes in each group (baseline *vs.* follow-up) using uncorrected statistical analyses to visualize the direction of changes.
Supplementary Figure 3 Pairwise group comparisons of longitudinal local gyrification index (LGI) changes.
Supplementary Figure 4 Pairwise group comparisons of longitudinal local gyrification index (LGI) changes including the parental education as one of the covariates.
Supplementary Figure 5 Pairwise group comparisons of longitudinal local gyrification index (LGI) changes including the illness duration at baseline as one of the covariates.
Supplementary Figure 6 Pairwise group comparisons of longitudinal local gyrification index (LGI) changes including the education as one of the covariates.
Supplementary Figure 7 Pairwise group comparisons of longitudinal local gyrification index (LGI) changes between the schizophrenia group who took typical antipsychotics and those took atypical antipsychotics.

Supplementary Table 1. Absolute local gyrification index (LGI) values in each time point and percent LGI change at the clusters showing significant differences in pairwise group comparisons of longitudinal LGI changes.

Cluster No.	Cluster Name (Region of intererst)	HC (*n* = 39) (Male 22, Female 17)		SzTypal (*n* = 14) (Male 10, Female 4)		Sz (*n* = 23) (Male 15, Female 8)		Analysis of covariance					
								Group		Time		Group × Time	
		mean	SD	mean	SD	mean	SD	F	*p*	F	*p*	F	*p*
1	Left rostral middle frontal							1.30	0.26	4.86	0.03	3.73	0.06
	Baseline	3.64	0.18	-	-	3.63	0.15						
	Follow-up	3.64	0.17	-	-	3.57	0.17						
	% Change	-0.04	2.94	-	-	-1.59	2.91						
2	Left superior frontal							0.04	0.84	5.29	0.02	2.87	0.10
	Baseline	1.91	0.09	-	-	1.94	0.07						
	Follow-up	1.91	0.08	-	-	1.90	0.08						
	% Change	-0.21	3.31	-	-	-1.73	3.54						
3	Right superior temporal							0.91	0.34	2.20	0.14	0.13	0.72
	Baseline	3.14	0.12	-	-	3.12	0.12						
	Follow-up	3.12	0.19	-	-	3.08	0.12						
	% Change	-0.66	5.48	-	-	-1.11	3.24						
4	Right precentral							0.11	0.74	0.00	0.96	0.00	0.99
	Baseline	3.24	0.26	-	-	3.23	0.24						
	Follow-up	3.24	0.31	-	-	3.22	0.27						
	% Change	0.46	11.67	-	-	0.52	12.03						
5	Left frontal pole							4.01	0.05	0.71	0.40	3.31	0.07
	Baseline	2.07	0.08	2.11	0.09	-	-						
	Follow-up	2.06	0.08	2.14	0.09	-	-						
	% Change	-0.43	3.42	1.35	2.57	-	-						
6	Right supramarginal							5.41	0.03	0.41	0.52	0.61	0.44
	Baseline	-	-	3.29	0.19	3.18	0.27						
	Follow-up	-	-	3.30	0.19	3.13	0.21						
	% Change	-	-	0.39	6.98	-1.37	6.87						

Abbreviations: HC, healthy control; LGI, local gyrification index; SzTypal, schizotypal; Sz, schizophrenia

Each LGI value was extracted from 6 clusters with significant differences in pairwise group comparisons of longitudinal LGI changes (Sz *vs*. HC, SzTypal *vs*. HC, and Sz *vs*. SzTypal) (Table 2, Figure 3). The percent LGI changes were calculated as follows: [(LGI value at follow-up – LGI value at baseline) / LGI value at baseline] × 100.

We compared the LGI changes between the pairwise groups using a repeated measure analysis of variance (ANCOVA), with group as a between-subject variable, time of testing (baseline, follow-up) as a within-subject variable, and age at baseline, sex, inter-scan interval, and cumulative medication dosage during the follow-up period as covariates.

Supplementary Table 2. Absolute local gyrification index (LGI) values in each time point and percent change at the clusters showing significant differences in pairwise group comparisons of longitudinal changes between combined patient group and healthy controls

Cluster No.	Cluster name	HC (Male 22, Female 17)		Combined group (Male 25, Female 12)		Analysis of covariance					
						Group		Time		Group x time	
	(Region of interest)	mean	SD	mean	SD	F	*p*	F	*p*	F	*p*
1	Left superior frontal					0.58	0.447	0.005	0.945	0.776	0.381
	Baseline	1.99	0.09	2.01	0.07						
	Follow-up	1.98	0.08	2.01	0.08						
	%Change	-0.46	3.49	-0.43	3.67						
2	Left caudal middle frontal					0.55	0.46	0.568	0.454	0.014	0.905
	Baseline	2.99	0.15	3.01	0.13						
	Follow-up	2.99	0.13	2.97	0.14						
	%Change	0.01	3.17	-1.35	2.93						
3	Left pars opercularis					0.19	0.67	0.437	0.511	1.713	0.195
	Baseline	4.27	0.26	4.28	0.32						
	Follow-up	4.29	0.28	4.25	0.29						
	%Change	0.44	3.62	-0.42	4.87						
4	Right precentral					0.05	0.82	0.021	0.884	0.272	0.603
	Baseline	3.22	0.23	3.23	0.25						
	Follow-up	3.21	0.27	3.22	0.28						
	%Change	0.28	9.56	0.03	10.29						

Abbreviations: HC, healthy control; LGI, local gyrification index

Each LGI value was extracted from 4 clusters with significant differences in pairwise group comparisons of longitudinal LGI changes (Combined patient group *vs*. HC) (Supplementary Figure 3). The percent LGI changes were calculated as follows: [(LGI value at follow-up – LGI value at baseline) / LGI value at baseline] × 100.

We compared the LGI changes between the pairwise groups using a repeated measure analysis of variance (ANCOVA), with group as a between-subject variable, time of testing (baseline, follow-up) as a within-subject variable, and age at baseline, sex, inter-scan interval, and cumulative medication dosage during the follow-up period as covariates.

Supplementary Figure 1 Longitudinal local gyrification index (LGI) changes in each group (baseline *vs.* follow-up) using uncorrected statistical analyses to visualize the direction of changes.

The schizophrenia (Sz) group (b) broadly exhibited a progressive decline in LGI in fronto-temporal areas, whereas the healthy control (HC) (a) and schizotypal (SzTypal) (c) groups showed increases in LGI over time. Brain regions with LGI increases were the fronto-temporal areas in the HC group (a), and the fronto-temporal, parietal, and occipital areas in the SzTypal group (c).

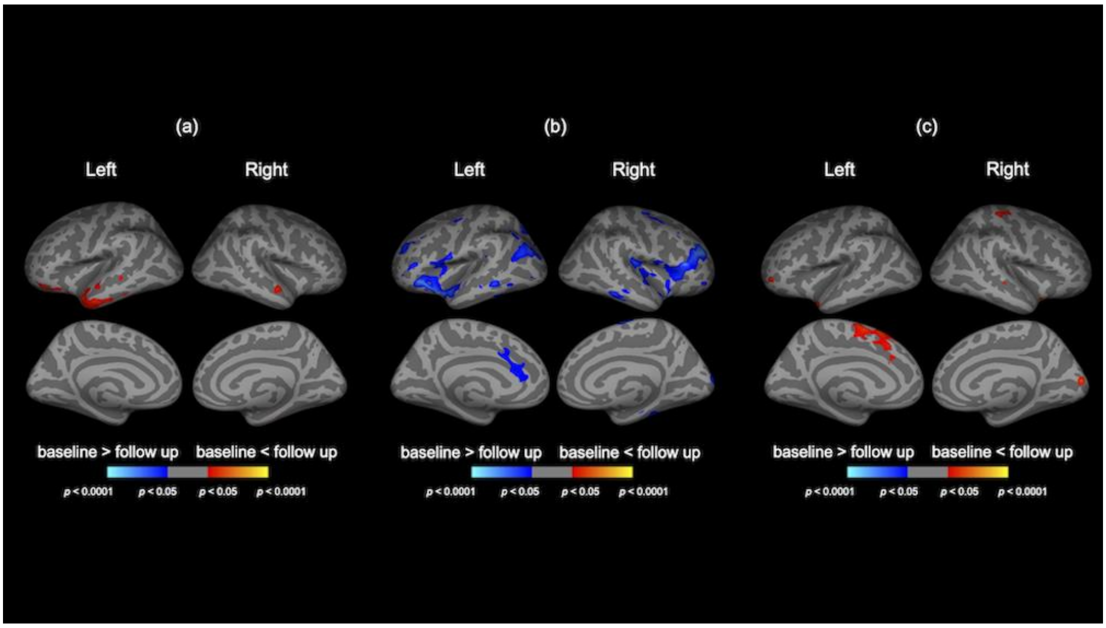

Supplementary Figure 2 Longitudinal local gyrification index (LGI) changes in each group (baseline *vs.* follow-up) using uncorrected statistical analyses to visualize the direction of changes.

The Combined patient group broadly exhibited a progressive decline in LGI in fronto-temporal areas (a).

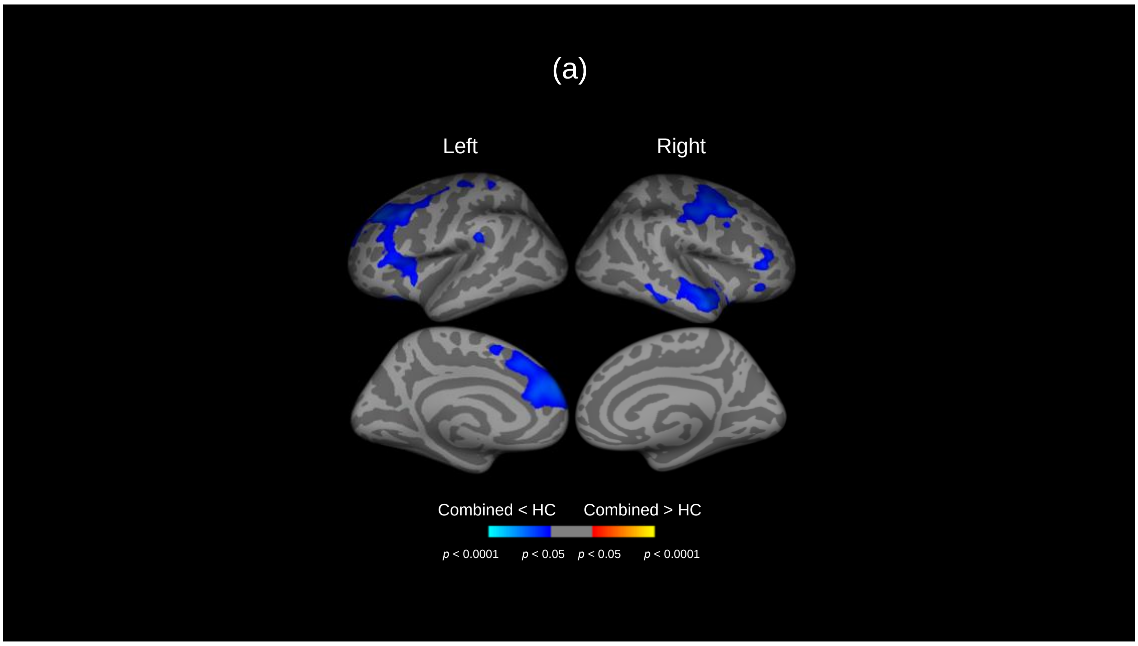

Supplementary Figure 3 Pairwise group comparisons of longitudinal local gyrification index (LGI) changes.

Cortical statistical maps showed that the Combined patient group exhibited a significantly greater decline in LGI over time than the healthy control (HC) group in the caudal middle frontal gyrus, superior frontal gyrus, and pars opercularis gyrus bilaterally, in addition to the rostral middle frontal gyrus, pars triangularis gyrus, and caudal anterior cingulate cortex in the left hemisphere (a).

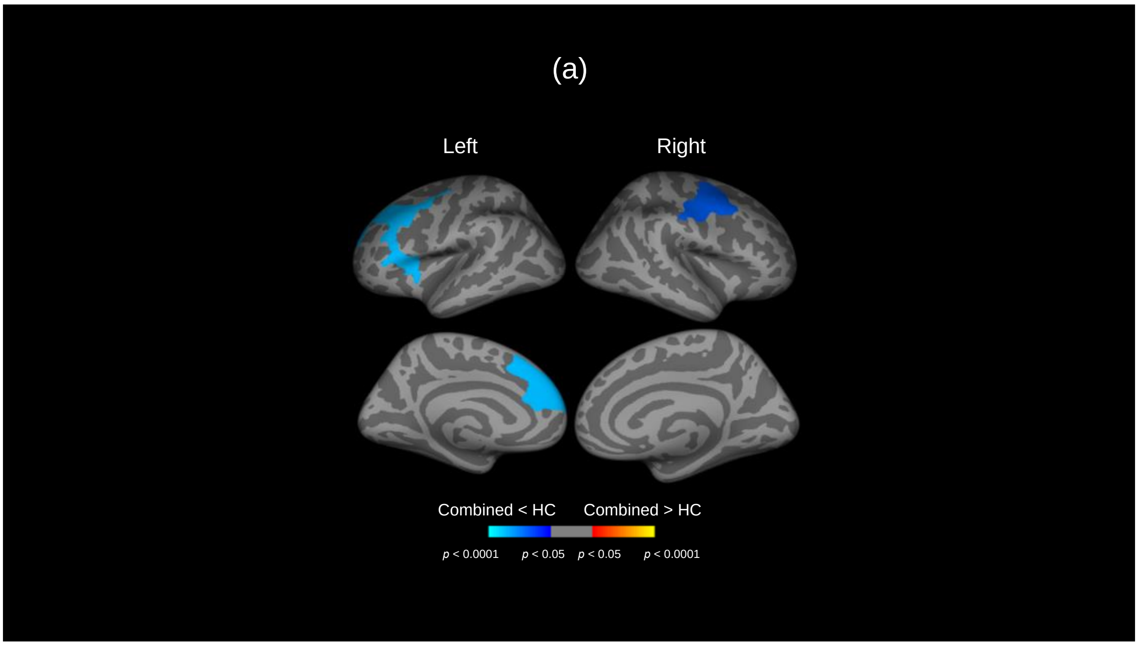

Supplementary Figure 4 Pairwise group comparisons of longitudinal local gyrification index (LGI) changes including the parental education as one of the covariates.

Cortical statistical maps showed that the schizophrenia (Sz) group exhibited a significantly greater decline in LGI over time than the healthy control (HC) group in the caudal middle frontal gyrus, superior frontal gyrus, and pars opercularis gyrus bilaterally, in addition to the rostral middle frontal gyrus, pars triangularis gyrus, and caudal anterior cingulate cortex in the left hemisphere, and superior temporal gyrus, precentral gyrus in the right hemisphere (a). The progressive increase in LGI in the left superior frontal area was smaller in the schizotypal (SzTypal) group than in the HC group (b). There were no significant differences in LGI changes in all pairwise group comparisons. The Sz group showed no significant LGI decline from the SzTypal group (c).

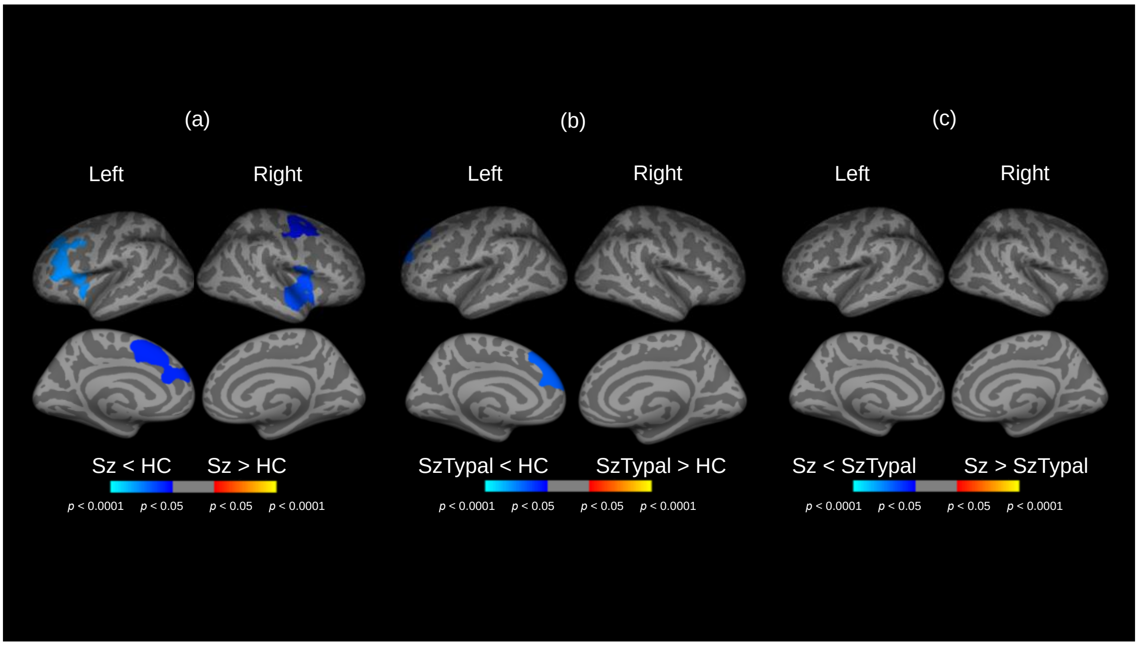

Supplementary Figure 5 Pairwise group comparisons of longitudinal local gyrification index (LGI) changes including the illness duration at baseline as one of the covariates.

Cortical statistical maps showed that the schizophrenia (Sz) group exhibited a significantly greater decline in LGI over time than the healthy control (HC) group in the caudal middle frontal gyrus and superior frontal gyrus in the left hemisphere as well as in the frontal pole, superior frontal gyrus, and medial orbitofrontal cortex in the right hemisphere. On the other hand, Sz group exhibited a significantly greater increase in LGI over time than the HC group in the lateral occipital cortex, isthmus cingulate cortex, cuneus gyrus, pericalcarine gyrus, lingual gyrus, and parahippocampal gyrus in the right hemisphere (a). The decline in LGI in the left superior frontal gyrus, left medial orbitofrontal cortex, right isthmus cingulate cortex, and right cuneus gyrus was greater in the schizotypal (SzTypal) group than in the Sz group, whereas those in the right frontal pole, right superior frontal gyrus, and right medial oribito-frontal gyrus was greater in the Sz group than in the SzTypal group (b).

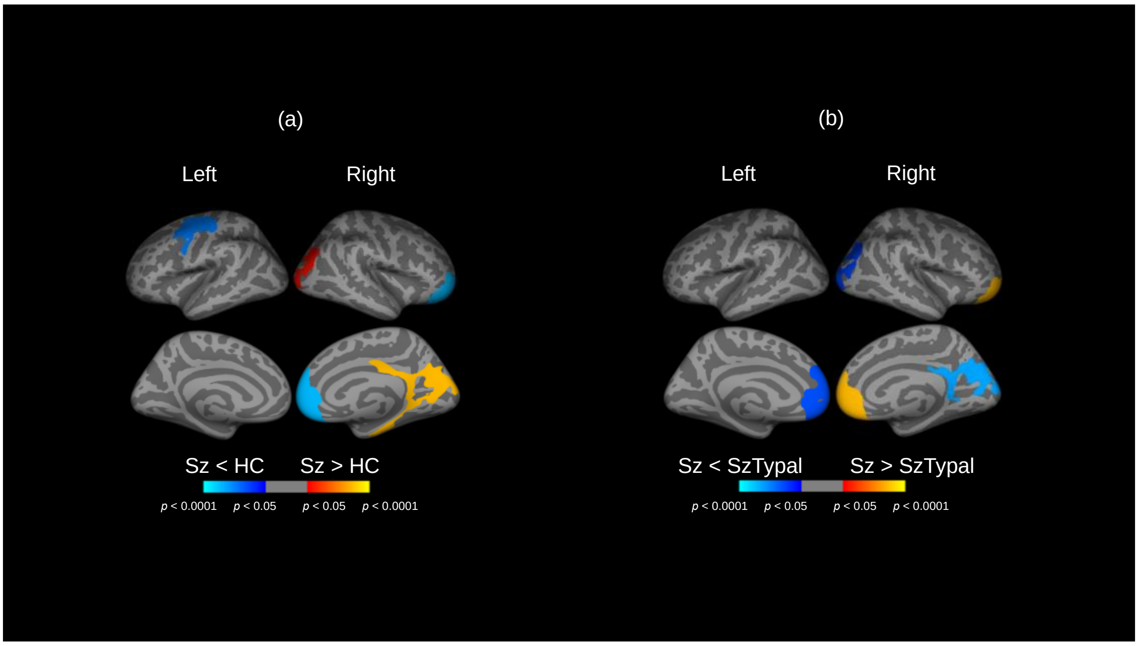

Supplementary Figure 6 Pairwise group comparisons of longitudinal local gyrification index (LGI) changes including the education as one of the covariates.

There were no significant differences in LGI changes in all pairwise group comparisons [Sz *vs.* HC (a), SzTypal *vs.* HC (b), and Sz *vs.* SzTypal (c)].

Abbreviation: HC, healthy control; Sz, schizophrenia; SzTypal, schizotypal

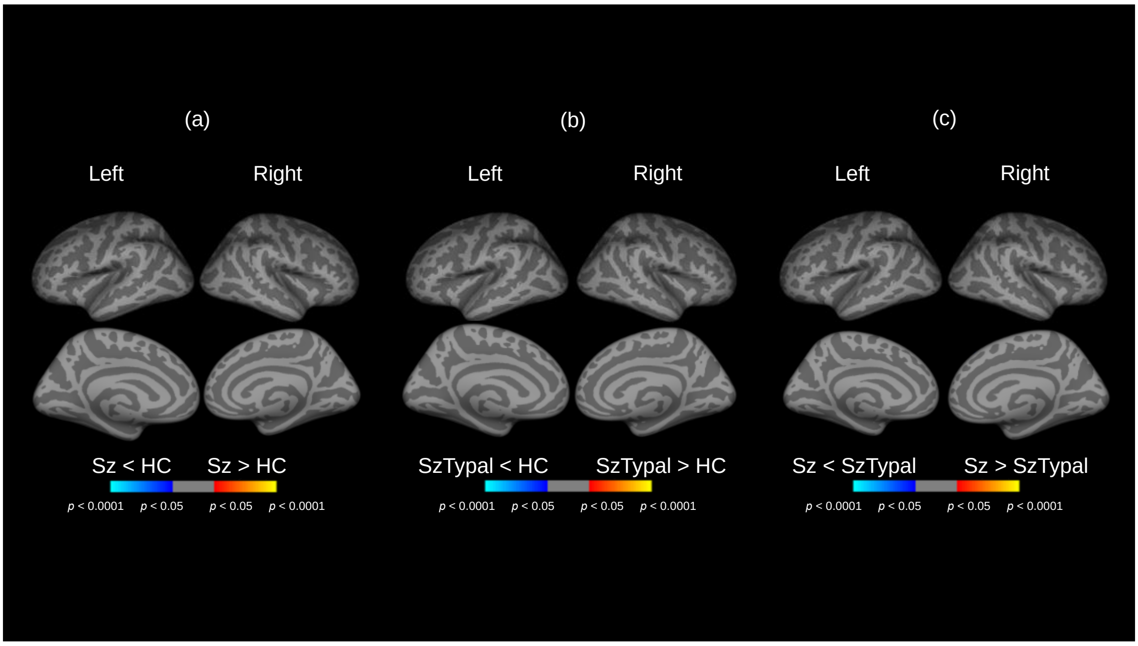

Supplementary Figure 7 Pairwise group comparisons of longitudinal local gyrification index (LGI) changes between the schizophrenia group who took typical antipsychotics and those took atypical antipsychotics.

Cortical statistical maps showed that the schizophrenia subgroup took typical antipsychotics (n = 7) group exhibited a significantly greater decline in LGI over time than the healthy control (HC) group in the caudal middle frontal gyrus, superior frontal gyrus, and pars opercularis gyrus bilaterally, in addition to the rostral middle frontal gyrus and pars triangularis gyrus in the left hemisphere (a). The schizophrenia subgroup took atypical antipsychotics (n = 16) group exhibited a significantly greater decline in LGI over time than the HC group in the rostral middle frontal gyrus, pars triangularis gyrus, and superior parietal cortex in the left hemisphere as well as in the superior temporal gyrus and precentral gyrus in the right hemisphere (b).

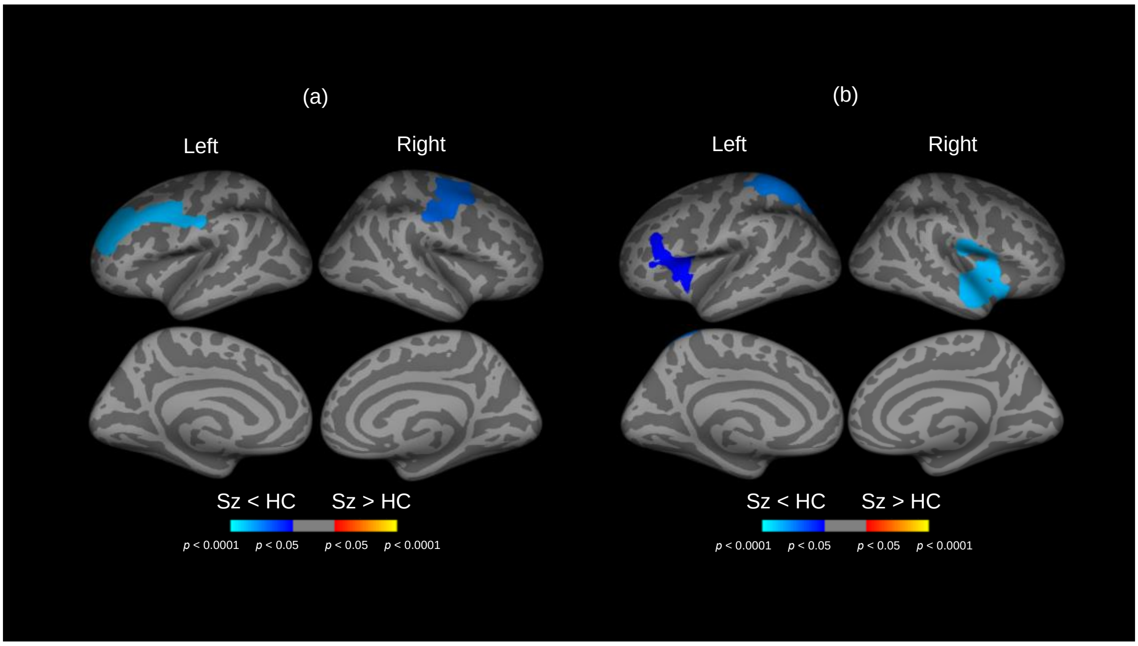

Supplement: Supplementary file 1 [file Data_Sheet_1.PDF]
